# Clinicopathological and prognostic significance of Twist overexpression in NSCLC

**DOI:** 10.18632/oncotarget.24489

**Published:** 2018-02-14

**Authors:** Meng Li, Xing Zhang, Xiaoqing Xu, Jiubin Wu, Kaiwen Hu, Xiuwei Guo, Peitong Zhang

**Affiliations:** ^1^ Department of Oncology, Guang’anmen Hospital, China Academy of Chinese Medical Sciences, Beijing, China; ^2^ Beijing University of Chinese Medicine, Beijing, China; ^3^ Department of Traumatology and Orthopedics, First Affiliated Hospital of Tianjin University of TCM, Tianjin, China; ^4^ Department of Oncology, Dongfang Hospital, Beijing University of Chinese Medicine, Beijing, China

**Keywords:** non-small cell lung cancer, Twist, prognosis, meta-analysis

## Abstract

Several studies were conducted to explore the prognostic significance of Twist in non-small cell lung cancer (NSCLC), however, contradictory results in different studies were reported. To this end, we presented a systematic review aiming to summarize the prognostic significance of Twist in patients with NSCLC. 5 studies involving a total of 572 patients were identified. The result indicated that high Twist expression was significantly associated with a worse overall survival (OS) (hazard ratio (HR) = 2.19, 95% confidence interval (95% CI) = 1.64–2.94, *p* < 0.001; I^2^ = 0.0%, fixed effect), recurrence-free survival (RFS) (HR = 2.476, 95% CI = 1.728–3.547, *p* < 0.001; I^2^ = 0.0%, fixed effect) and lymph node or other metastasis (odds rate (OR) = 0.419, 95% CI = 0.259–0.679, *P* < 0.001, fixed effect). Subgroup analysis revealed that the expression of Twist in Chinese patients might be more closely associated with the prognosis of NSCLC than in American patients. Overall, these results indicated that Twist over-expression in patients with NSCLC might be related to poor prognosis and serves as an unfavorable predictor of poor clinicopathological prognosis factor.

## INTRODUCTION

Primary lung cancer is the leading cause of cancer mortality worldwide among both men and women [1, 2], and non-small cell lung cancer (NSCLC) accounts for about 80% of all lung cancer [3]. The high recurrence and metastasis largely contribute to the poor overall prognosis [4]. Thus, the lack of major improvement in the 5-year survival rate of NSCLC has driven the search for new strategies aiming at identifying specific biological markers of metastases to predict the prognosis in patients.

Local invasion can be considered as an initial step in the malignancy of carcinomas, leading to the generation of distant metastasis. Epithelial to mesenchymal transition (EMT) is an epithelial cell into a mesenchymal cell with the ability to invade and migrate adjacent tissues. It is a recent discovered cellular process in a wide variety of cancer cells, and it enhances the potential of tumor invasion, metastasis and chemoresistance [5, 6]. One of the hallmarks of EMT is the functional loss of E-cadherin, which is currently thought to be a suppressor of invasion during carcinoma progression [7]. Indeed, transcriptional repression has emerged as a fundamental mechanism for repressing E-cadherin during tumour progression. As one of multifunctional transcription factors that dynamic silences E-cadherin, Twist is now thought to be involved in tumour progression, thus having potential clinical interest [8, 9]. The two Twist-like proteins, Twist-1 and Twist-2 (formerly Dermo-1), belong to the basic helix-loop-helix (bHLH) family and have been identified to promote EMT [10–13]. Well conserved during evolution, two Twist genes exist in vertebrates and share high structural homology. [14] These two proteins display a high degree of sequence similarity in their C-terminal half and are more divergent in their N terminus. When Twist-2 lacks a glycine-rich region that presents in Twist-1 [15]. While both are silent in most healthy adult tissues, they were found overexpressed in various types of human tumors including a variety of carcinomas as well as sarcomas, melanomas, gliomas and neuroblastomas [16]. Twist-1 and Twist-2 do not display major transforming capabilities but still cooperate with activated oncoproteins in promoting EMT. [14] In the course of tumor progression, reactivation of EMT inducing transcription factors, Twist-1 and Twist-2, trigger the generation of cells with self-renewal capabilities, favoring both the growth of the primary tumor and the initiation of secondary tumors following cell dissemination. While, in most articles, Twist is also known as Twist-1 [9]. Although Twist-1/2 was included when literature searching in this article, actually, Twist-1 was discussed by carefully reading the finally involved five articles. Twist-1(Twist) overexpression has been reported in a variety of epithelial cancer cells with clinical correlations with poor prognosis, an event associated with its capacity to promote EMT [16–19] and these malignancies include breast cancer [20], colorectal cancer [21], pancreatic ductal adenocarcinoma [22] and oral cancer [23]. However, the prognostic value of Twist in lung cancer remains controversial. Some studies [24–26] have suggested that the expression of Twist might not predict the survival of NSCLC, while the other researches [27–30] have indicated a specific correlation between Twist expression and patient prognosis. Besides, the research of Hung et al. [31] suggested Twist was associated with a shorter overall survival (OS), whereas the overexpression of Twist did not influence recurrence-free survival (RFS). We therefore carried out a systematic review of published literatures to quantitatively analyze the prognostic value of Twist in NSCLC patients based on current evidence.

## RESULTS

### Literature search

**Figure 1:**
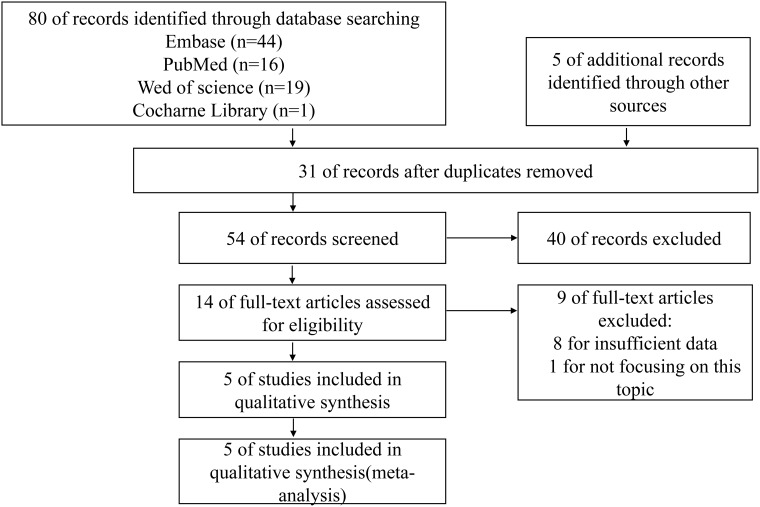
Flow diagram of study selection process

The literature search strategy and study selection are summarized in Figure 1. A total of 85 papers were identified of which 31 duplicative papers were excluded. As for the remaining 54 papers, 40 were excluded by scanning either the title or abstract. For the 14 remaining potentially related studies, the full-text was carefully read. 8 were excluded for insufficient data to assess the hazard ratios (HRs) of prognosis outcomes, and 1 was excluded for not focusing on this topic. Although evaluating the estimated HRs from the survival curves might be a reliable and the only feasible approach [32, 33], however, we found major deviations in two papers [34, 35] extracted from the same study of Pallier K et al. [36]. Thus, this study [36] failed to directly reveal the HRs and 95% confidence interval (95% CI) from the original data was excluded. At last, 5 studies involving 572 patients were eligible for this meta-analysis [27–31].

### Characteristic of included studies

As listed in Table 1, the five included studies contained 572 patients. As for assessment of included studies, the Newcastle-Ottawa Quality Assessment Scale (NOS) of four included studies was 7 and one included study was 6 (Supplementary Table 1). Out of the five studies, four evaluated patient cohorts proceeded in China, and one was from America. Three studies applied OS and RFS as outcomes to explore prognosis correlation with Twist expression; the other two studies considered OS as the only outcome. Three studies demonstrated that Twist overexpression was an independent prognostic factor (*P* < 0.05) [27, 28, 30], while Hui et al. [29] suggested an inverse correlation between Twist expression and patient prognosis by a multivariate Cox regression analysis (*P* > 0.05). Besides, another study [31] suggested Twist was associated with a shorter OS instead of RFS. Four studies reported the follow-up time (range, 3 to 95 months), while the other one did not report the follow-up time [27]. In addition, the sample size was different, varying from 75 patients to 153 patients.

**Table 1:** Characteristics of the included studies

study	Country	Sample size (*n*)	Histology type (AC%)	Twist positive (%)	Outcome	Cut-off	Analysis	Follow-up (M)	recruitment time
Hung, J. J, 2009	China	87	62.10%	36.8	RFS/ OS	Positive > 50%	U	Median43.2 (20 to 52.2)	2003–2004
Jiang W, 2012	China	137	59.90%	40	RFS/ OS	Positive > 60%	U	Median 39 (24.9 to 49.3)	2006–2010
Hui LP, 2013	China	120	47.50%	38.3	OS	Normal: 0-1; overexpression: 2-9	M	Median 30.8 (3 to 72)	2001–2010
Lv TF, 2015	America	75	96.00%	34.7	OS	low: ≤ 1; overexpression: ≥ 2	NM	NM	2004–2009
Zhou Y, 2016	China	153	17.00%	78.4	OS	low:< 4; overexpression: ≥ 4	U / M	Median 57(4 to 95)	2002–2004

RFS recurrence-free survival; AC adenocarcinoma; U univariate analyses; M multivariate analyses; NM no mention.

### Association of twist with survival

### Meta-analysis of OS

**Figure 2:**
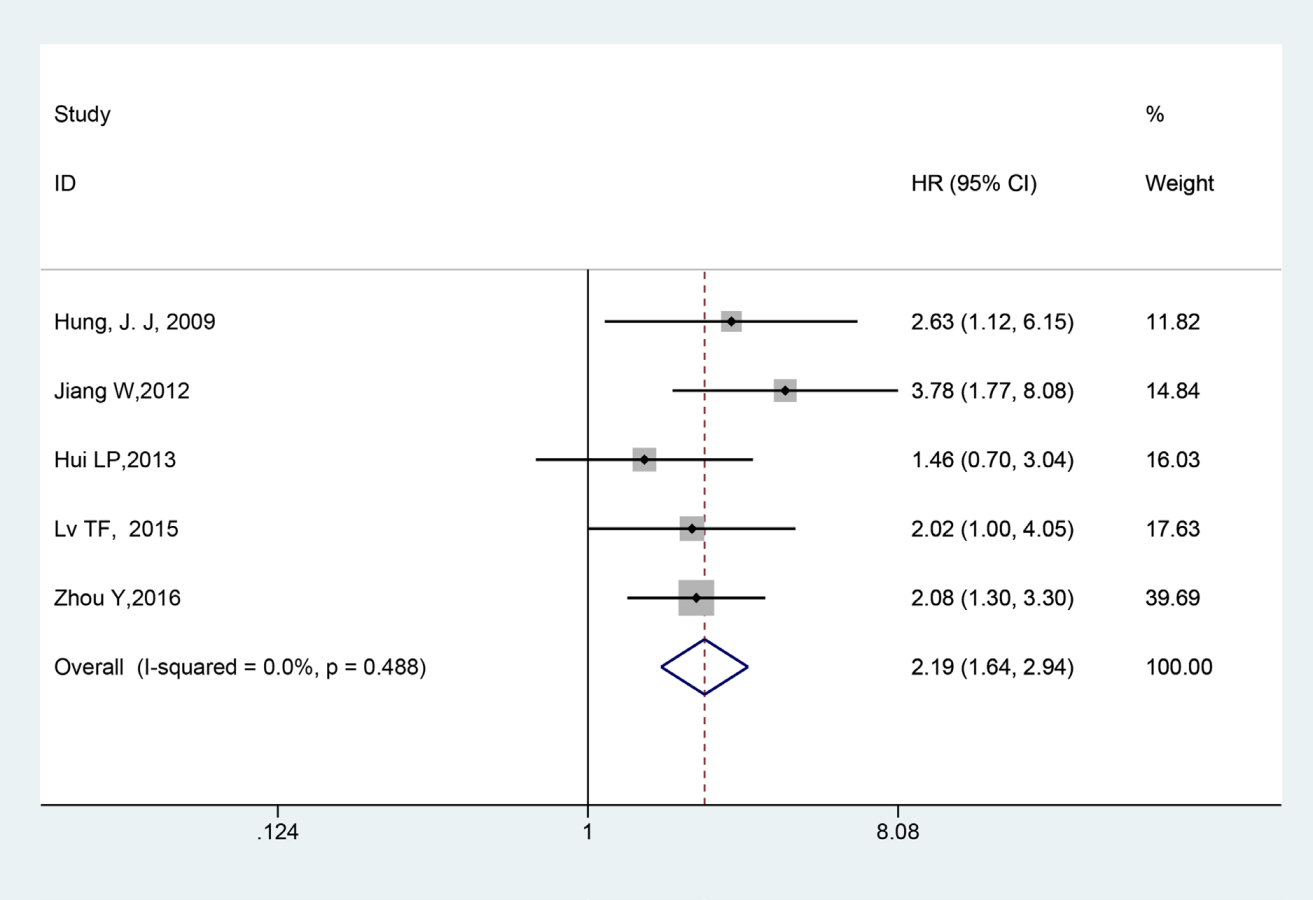
Forest plot of the correlation between twist and OS in NSCLC patients

Five studies involving 572 patients were included in the meta-analysis of OS. As shown in Figure 2, in view of the significant heterogeneity (I^2^ = 0.0%, *p* = 0.488), the fixed-effect model was used. A significant correlation between the expression of Twist and OS was observed (HR = 2.19, 95% CI = 1.64–2.94, *p* < 0.001), and the result revealed that overexpression of Twist predicted worse OS when compared with the low expression of Twist.

### Subgroup meta-analyses

**Figure 3:**
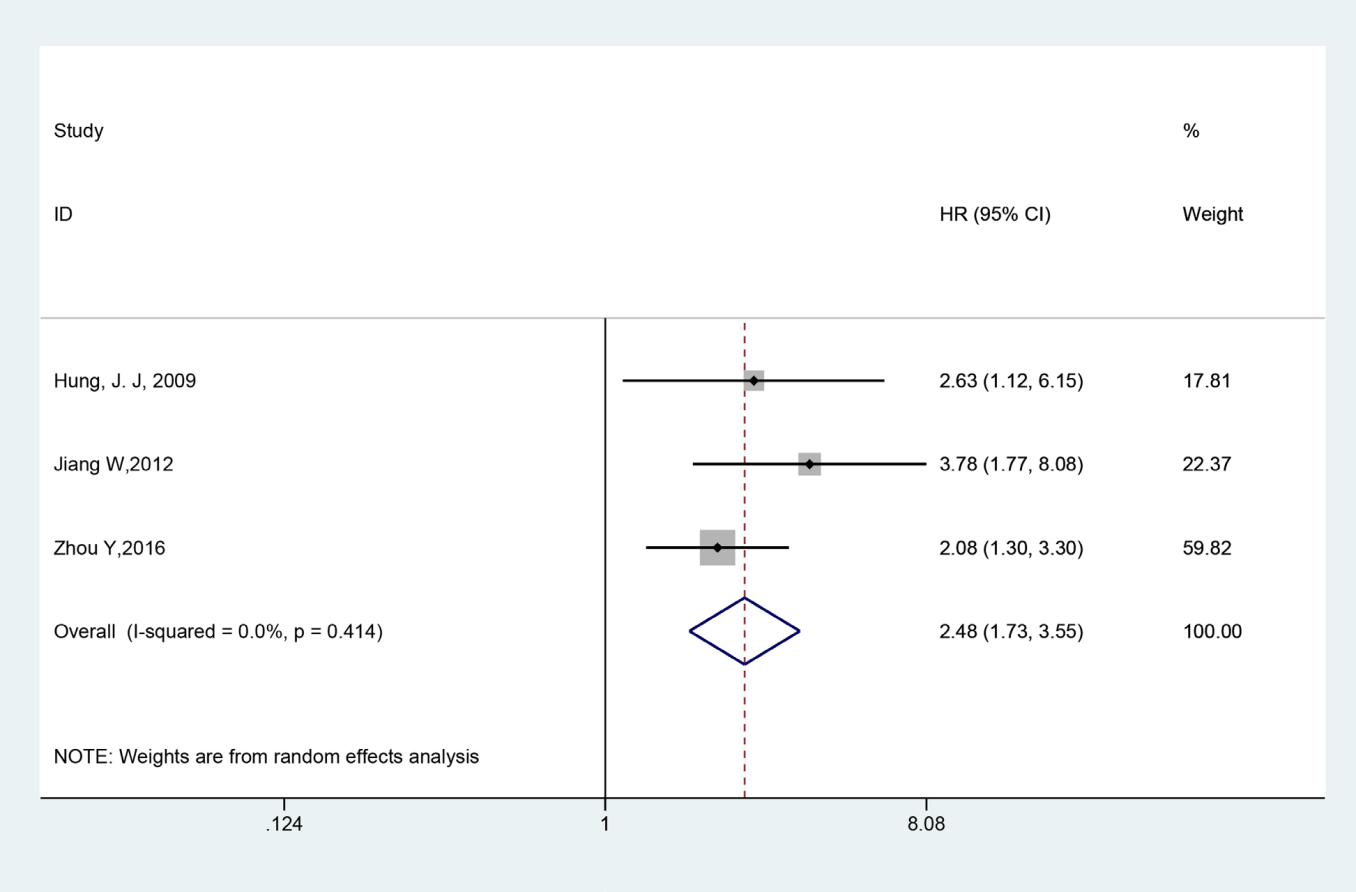
Forest plot of the correlation between twist and RFS in NSCLC patients

Table 2 shows the subgroup meta-analyses. All pooled HRs were obtained by using a fixed-effect model. Three studies reporting the RFS of patients with NSCLC were all included into the meta-analysis. As shown in Figure 3 and Table 2, a distinct correlation was observed between the Twist and RFS (HR = 2.476, 95% CI = 1.728–3.547, *p* < 0.001), with heterogeneity I^2^ = 0.0% (*p* = 0.414). Poor prognosis was found in NSCLC with Twist overexpression under univariate analyses (pooled HR = 3.219, 95% CI = 1.826–5.674, *p* < 0.001) and multivariate analyses (pooled HR = 1.877, 95% CI = 1.268–2.779, *p* = 0.002). Results showed that in terms of country, unfavorable prognosis was found in China (pooled HR = 2.235, 95% CI = 1.619–3.086, *p* < 0.001). Among the study with follow-up, unfavorable survival results were obtained whether the follow-up time was longer than 36 months or not (Follow-up (month) > 36, pooled HR = 2.476, 95%CI = 1.728–3.547, *P* < 0.001; Follow-up (month) < 36/no mention, pooled HR = 1.731, 95% CI = 1.045–2.866, *P* = 0.033).

**Table 2:** Meta-analysis of twist overexpression and prognosis in NSCLC

Categories	Studies (patients)	HR (95% CI)	I^2^ (%)	P_H_	Z	P
RFS	3 (377)	2.476 (1.728–3.547)	0.0	0.414	4.94	< 0.001
Univariate analyses	2 (224)	3.219 (1.826–5.674)	0	0.533	4.04	< 0.001
Multivariate analyses	2 (273)	1.877 (1.268–2.779)	0	0.429	3.15	0.002
Country (China)	4 (497)	2.235 (1.619–3.086)	10.8	0.339	4.89	< 0.001
Follow-up (M) > 36	3 (410)	2.476 (1.728– 3.547)	0	0.414	4.94	< 0.001
Follow-up (M) < 36/NM	2 (162)	1.731 (1.045– 2.866)	0	0.534	2.13	0.033

All pooled HRs were performed by fixed-effect model.

P_H_ *P*-value for heterogeneity based on *Q* test.

P *P*-value for statistical significance based on *Z* test.

### Association of twist with clinicopathological parameters

**Figure 4:**
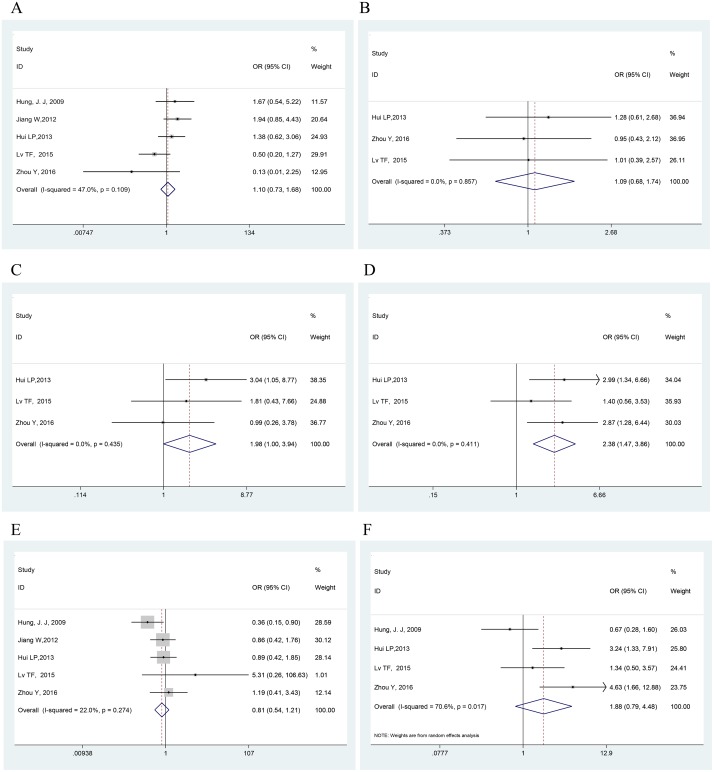
Forest plots showing the OR of Twist overexpression vs. normal Twist expression for clinicopathological features (**A**) Age; (**B**) Sex; (**C**) Tumor differentiation; (**D**) Lymph node or other metastasis; (**E**) Histology; (**F**) Tumor stage. All pooled ORs were performed by fixed-effect model except for tumor stage with (NOTE: Weights are from random effects analysis). The difference between Twist overexpression and lymph node or other metastasis was statistically significant (OR = 2.384, *P* < 0.001).

The connections between Twist and clinicopathological parameters are shown in Table 3 and Figure 4. The difference between Twist overexpression and biologically aggressive phenotypes, such as lymph node or other metastasis (OR = 2.384, 95% CI = 1.472–3.862, *P* < 0.001, fixed effect) was statistically significant. However, no association was found between Twist and other clinicopathological features, including age (OR = 1.086, 95% CI = 0.679–1.736, *P* = 0.731, fixed effect), sex (OR = 1.104, 95% CI = 0.726–1.679, *P* = 0.644, fixed effect), tumor differentiation (OR = 1.981, 95% CI = 0.996–3.939, *P* = 0.051, fixed effect), histology type (OR = 0.810, 95% CI = 0.544–1.206, *P* = 0.299, fixed effect) and tumor stage (OR = 1. 883, 95% CI = 0.791–4.485, *P* = 0.153, random effect).

**Table 3:** Meta-analysis of Twist overexpression and clinicopathological features in NSCLC

Categories	Studies (patients)	OR (95% CI)	I^2^ (%)	P_H_	Z	P
Age (> 60/≤ 60)	3 (348)	1.086 (0.679–1.736)	0	0.857	0.34	0.731
Sex (Male / Female)	5 (572)	1.104 (0.726–1.679)	47.0	0.109	0.46	0.644
Tumor differentiation (Moderate, Poor / Well)	3 (348)	1.981 (0.996–3.939)	0	0.435	1.95	0.051
Lymph node or other metastasis	3 (348)	2.384 (1.472–3.862)	0	0.411	3.53	< 0.001
Histology (adenocarcinoma/non-adenocarcinoma)	5 (572)	0.810 (0.544–1.206)	22.0	0.274	1.04	0.299
Tumor stage (II, III, IV/I)	4 (435)	1. 883 (0.791–4.485)	70.6	0.017	1.43	0. 153

All pooled ORs were performed by fixed-effect model except for tumor stage with random-effect model.

OR odds rate

P_H_ *P*-value for heterogeneity based on *Q* test.

P *P*-value for statistical significance based on *Z* test.

### Sensitivity analysis and publication bias

**Figure 5:**
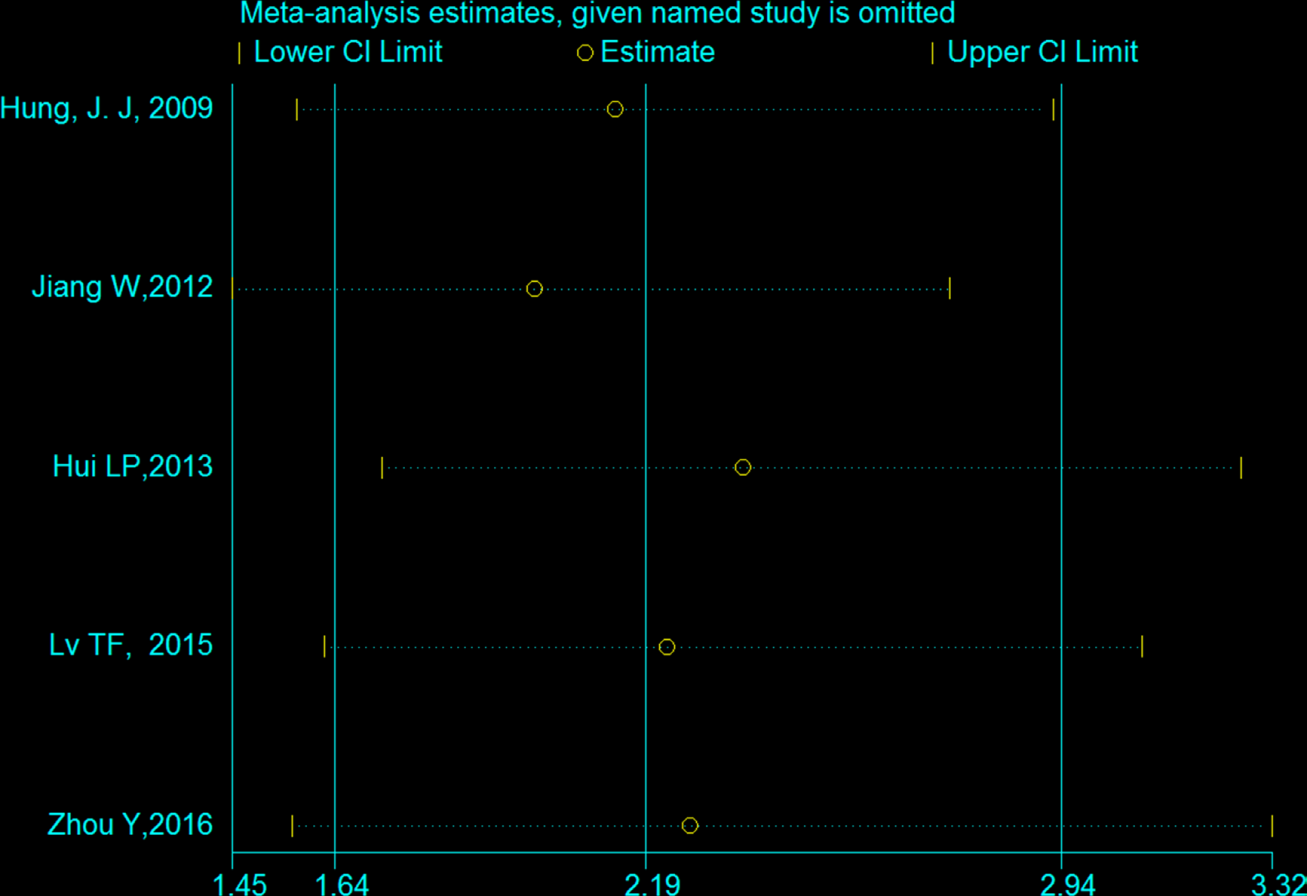
Effect of individual studies on the pooled HR for Twist overexpression and OS of NSCLC The horizontal axis number 2.19 represents the overall HR, and the 1.64 and 2.94 represent the 95% CI.

**Figure 6:**
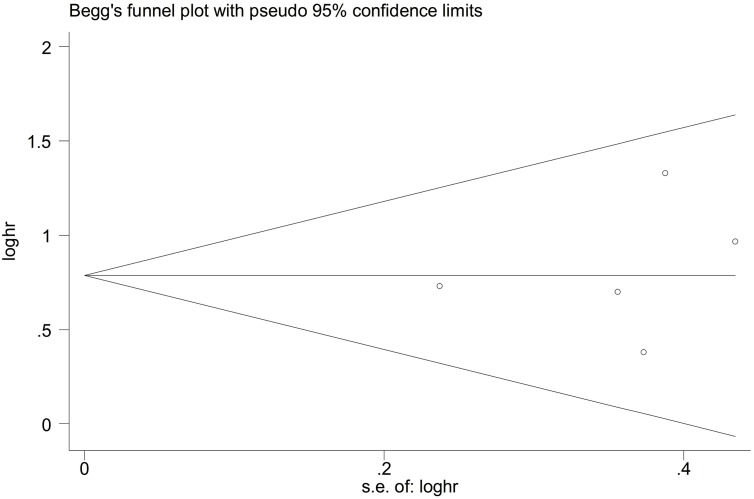
Funnel plot analysis to detect publication bias There was no publication bias (*P* = 0.462).

The result of the sensitivity analysis is shown in Figure 5. When each independent study was sequentially excluded, the combined 95% CI of the remaining four studies did not exceed the 95% CI of the pooled HR of five studies, indicating that no study dominated the result. Publication bias was tested by HR estimation of the OS. No obvious publication bias was revealed by Begg's test (*P* = 0.462) and Egger's test (*P* = 0.651) (Figure 6).

## DISCUSSION

NSCLC is the most predominant type of lung cancer and the leading cause of cancer death worldwide. However, there is little by using a combination of metastasis-related markers. EMT is considered to be one of the major molecular mechanisms inducing tumour invasion and metastasis. Twist is a highly conserved bHLH transcription factor that had been found to induce cancers promoting through EMT. There is increasing evidence to support the expression of Twist is associated with worse survival in carcinoma patients, including lung cancer [35]. *In vivo* and *in vitro* studies were implemented to gain a comprehensive analysis of Twist in NSCLC patients, animal models, and human NSCLC cell lines. Twist and related signal transduction pathways play important roles in carcinoma progression and may serve as potential target for treating carcinoma [37]. Twist is a significant prognostic marker to predict overall survival in patients with lung adenocarcinoma [25]. The findings supported that Twist expression was linked to EGFR mutations by collaborating with the EGF pathway in promoting EMT in EGFR mutated lung adenocarcinoma [36]. Twist is associated with hypoxic metastasis and EMT of NSCLC and serves as a potential therapeutic target in hypoxic lung cancer [35, 38]. Moreover, Twist is correlated with other co-expression markers (p-4E-BP1, HIF-1α, Snail, N-cadherin, Foxc2, Slug) in predicting the worse prognostic outcome of NSCLC [27, 29–31]. Sufficient data have shown a significant correlation between Twist expression and poor prognosis of NSCLC patients. However, controversy exists concerning the correlation between Twist and prognostic value with respect to NSCLC.

In this study, we meta-analyzed the literature on Twist expression in NSCLC and its association with OS and clinicopathological features. Results showed that Twist overexpression was correlated with poor OS and RFS. All subgroup analysis demonstrated the adverse role of Twist overexpression in NSCLC. Moreover, the sensitivity analysis indicated that our results were relatively conclusive. Thus, Twist expression may be an independent prognostic factor of OS. We also found a significant association between Twist overexpression and poor clinicopathological features, such as metastasis including lymph node metastasis. This study is a literature-based analysis, Begg's funnel plot and Egger's test were used to assess publication bias. All five eligible studies yielded a Begg's test score of *P* = 0.462 and Egger's test score of *P* = 0.651. These results suggested that there was no publication bias.

Although this meta-analysis aimed to provide the best possible estimate of the clinical significance of Twist expression, it still had limitations. First of all, all the included studies were retrospective, which might increase the bias of our study. Secondly, the sample size of included articles was relatively small with only 572 cases. Thirdly, no methods and cutoff definitions had been accepted and validated for evaluating Twist expression. Finally, although publication bias was not occurred in this study, since most of including studies were from China, the researchers tended to publish unusually high proportion of positive results [39].

In conclusion, despite the limitations listed above, this meta-analysis showed that Twist overexpression is correlated with poor OS and clinicopathological features in NSCLC, suggesting that the Twist may be a poor prognostic factor and therapeutic target in NSCLC. Subgroup analysis revealed that the expression of Twist in Chinese patients may be more closely associated with the prognosis of NSCLC than in other countries. Moreover, further larger well-designed prospective studies employing a standard evaluated method are needed to confirm these findings from our study.

## MATERIALS AND METHODS

Meta-analysis was conducted in accordance with the preferred reporting items for meta-analysis criteria [40].

### Literature search strategy

We performed a complete computer-based search of the PubMed, Embase, web of science and the Cochrane Library databases for clinical trials in original articles up to the date of July 10, 2017. The search strategy was conducted according to a combination of the following terms: “((non-small cell lung cancer) OR NSCLC) AND ((Twist) OR TWIST) AND (((((prognosis) OR outcome) OR survival) OR prognostic value) OR prognostic biomarker)”. The irrelevant articles were directly excluded by scanning the titles or abstracts. We also examined reference lists from the adopted studies in order to avoid missing studies which may fulfill our requirements. The remaining articles were then reviewed comprehensively by reading the full text.

### Inclusion criteria

Studies meeting all the following criteria were included: 1) retrospective or prospective studies; 2) paying attention to the relationship between Twist expression and survival in NSCLC; 3) providing enough data to get the HR for prognosis outcomes, along with their 95% CIs or *P* values; 4) published in English.

### Exclusion criteria

The exclusion criteria were as followed: 1) neither the retrospective nor prospective studies; 2) studies without sufficient data to pool the HR; 3) studies not focusing on the role of the Twist expression on the prognosis in NSCLC; 4) not published in English; 5) were reviews, case reports, conference abstracts or comments, or studies *in vitro* or in animal models.

### Data abstraction

Two investigators (Meng Li and Zhang X) independently reviewed all the manuscripts. The following data were abstracted: first name of the author, publication year, country of the study, sample size, histology type, positive rate of Twist, outcome, cut-off value of Twist expression, survival analysis, follow-up time and recruitment time. The HRs of RFS or OS obtained directly from published articles. The HR assessed by multivariate analysis was abstracted when the multivariate analysis and univariate analysis were both provided. All HR and 95% CI were reported in these studies.

### Quality assessment

The Newcastle-Ottawa Quality Assessment Scale (NOS) was applied to assess the quality of the selected studies. The NOS included three main aspects: selection, comparability, and outcome [41, 42]. And NOS score ≥ 6 is considered high-quality study. Discrepancy was resolved by consulting.

### Statistical analysis

Pooled analyses were carried out by STATA software (version 14.0; StataCorp, College Station, Texas, USA). The prognosis outcomes were explored using the HR and the corresponding 95% CI. The prognosis outcomes mainly contained the OS or RFS. The heterogeneity was assessed by Cochran's *Q* test and Higgins I^2^ significant when *P* < 0.05 and/or I^2^ [43]. When the heterogeneity was > 50%, the random effect model was used; if not, the fixed-effects model was applied. Stata 14.0 was used to assess the robustness of the results. All the P was two-side and *P* < 0.05 was considered statistically significant. Visual inspection of Begg's test and Egger's test [44] were used to evaluate publication bias (*P* < 0.01 was considered statistically significant). The sensitivity analysis was conducted by using the “metaninf” STATA command (sequential exclusion of each individual study then pooled HR).

## SUPPLEMENTARY MATERIALS TABLE



## Abbreviations

NSCLC: non-small cell lung cancer
OS: overall survival
RFS: recurrence-free survival
95% CI: 95% confidence interval
EMT: Epithelial to mesenchymal transition
HR: hazard ratio
OR: odds rate
bHLH: basic helix-loop-helix
NOS: Newcastle-Ottawa Quality Assessment Scale
