# Supplementary material for: Clinicopathological and prognostic significance of Twist overexpression in NSCLC
Source: Oncotarget. 2018 Feb 14;9(18):14642–51. doi: 10.18632/oncotarget.24489 (PMC5865696; doi:10.18632/oncotarget.24489)
Supplement: Supplementary file 1 [file oncotarget-09-14642-s001.pdf]

Clinicopathological and prognostic significance of Twist overexpression in NSCLC

SUPPLEMENTARY MATERIALS

Supplementary Table 1: Assessment of study quality

study	Quality indicators from the Newcastle-Ottawa scale									Score
	Selection				Comparable		outcome assessment			
	1	2	3	4	5	6	7	8	9	
Hung et al. [27]	*	*	*		*	*	*	*		7
Jiang et al. [26]	*	*	*		*	*	*	*		7
Hui et al. [25]	*	*	*		*	*	*	*		7
Lv et al. [23]	*	*	*		*	*	*			6
Zhou et al. [24]	*	*	*		*	*	*	*		7

*For cohort studies, 1, indicates exposed cohort truly representative; 2, non-exposed cohort drawn from the same community; 3, ascertainment of exposure; 4, outcome of interest not present at start; 5, cohorts comparable on basis of age; 6, cohorts comparable on other factor(s); 7, quality of outcome assessment; 8, follow-up long enough for outcomes to occur; and 9, complete accounting for cohorts.
